# Auricular transcutaneous vagus nerve stimulation stabilizes event segmentation through modulation of working memory representations

**DOI:** 10.1093/ijnp/pyag002

**Published:** 2026-01-23

**Authors:** Xianzhen Zhou, Foroogh Ghorbani, Veit Roessner, Bernhard Hommel, Astrid Prochnow, Christian Beste

**Affiliations:** Cognitive Neurophysiology, Department of Child and Adolescent Psychiatry, Faculty of Medicine, TU Dresden, Dresden, Germany; Cognitive Neurophysiology, Department of Child and Adolescent Psychiatry, Faculty of Medicine, TU Dresden, Dresden, Germany; Cognitive Neurophysiology, Department of Child and Adolescent Psychiatry, Faculty of Medicine, TU Dresden, Dresden, Germany; German Center for Child and Adolescent Health (DZKJ), Partner Site Leipzig/Dresden, Dresden, Germany; Shandong Provincial Key Laboratory of Brain Science and Mental Health, Faculty of Psychology, Shandong Normal University, Jinan, China; Cognitive Neurophysiology, Department of Child and Adolescent Psychiatry, Faculty of Medicine, TU Dresden, Dresden, Germany; Cognitive Neurophysiology, Department of Child and Adolescent Psychiatry, Faculty of Medicine, TU Dresden, Dresden, Germany; German Center for Child and Adolescent Health (DZKJ), Partner Site Leipzig/Dresden, Dresden, Germany; Shandong Provincial Key Laboratory of Brain Science and Mental Health, Faculty of Psychology, Shandong Normal University, Jinan, China

**Keywords:** event segmentation, atVNS, GABA, norepinephrine

## Abstract

**Background:**

Segmenting continuous experience into discrete units—event segmentation—is fundamental for situational awareness and adaptive action. Based on Event Segmentation Theory, we used auricular transcutaneous vagus nerve stimulation (atVNS) to test whether the control of event segmentation is moderated by norepinephrine and/or GABAergic systems.

**Methods:**

Healthy adults (*N* = 40) completed a double-blind, counterbalanced experiment in which they watched a narrative movie and indicated perceived event boundaries under active atVNS and sham stimulation while the electroencephalogram (EEG) was recorded. Multivariate pattern analysis (MVPA) quantified the decodability of boundary interval (BI) vs no-boundary interval (NBI), and EEG source reconstruction assessed cortical activation.

**Results:**

Relative to sham, active atVNS reduced the likelihood of segmentation. Converging neurophysiological evidence mirrored this behavioral effect: MVPA revealed lower decoding accuracy for BI and NBI, indicating less distinct neural representational patterns. Source reconstruction showed reduced activation in the prefrontal cortex, a region involved in working memory.

**Conclusions:**

Across behavioral and neural measures, atVNS stabilized ongoing event representation and restricted the updating of the current event working model, consistent with a GABAergic modulation of event segmentation. These findings extend prior work linking atVNS to working memory gating and demonstrate its impact on an ecologically relevant cognitive process—event segmentation.

**Significance Statement**

This study tests whether 2 neuromodulators—norepinephrine (NE) and GABA—causally change how people divide continuous experience into meaningful events—a process called event segmentation. We used auricular vagus nerve stimulation (atVNS) to nudge these systems and compared active stimulation with a sham control. Compared to sham, active atVNS reduced the probability of setting event boundaries. In parallel, the brain activity patterns before the moments of segmenting were less distinct from the no-boundary period than in the sham condition, with lower activity in prefrontal regions. Together, these converging behavioral and neural findings indicate that atVNS stabilizes ongoing event representations and restricts the updating of the current event working model, a pattern that more parsimoniously supports a GABAergic inhibitory gating account over an NE arousal account of event segmentation.

## INTRODUCTION

Structuring the vast amount of incoming information from our environment is essential for orienting ourselves within it and for executing adaptive behavior. A key process in this regard is event segmentation, which has been conceptualized in the Event Segmentation Theory (EST).^1^^,^^2^ At the core of this theory lies the working event model, which integrates current perceptual information with prior experiences of similar situations—stored in long-term memory as so-called event schemata—into a coherent representation of the present situation.^1-3^ This representation can then be used to generate predictions about how the situation will unfold. If these predictions are violated, or if they correspond to a change deemed too substantial, an event auricular transcutaneous vagus nerve stimulation (atVNS) served to stabilize the boundary is established, triggering an update of the working event model.^1^^,^^2^ Thus, the working event model continuously balances maintaining existing information and updating when predictions fail, as an ongoing decision about whether the current deviation from prediction remains acceptable. Shifting between maintaining and updating of working memory representations—such as working event models—can be considered as working memory gating decisions: either open the gate to admit new information and trigger an update, or keep the gate closed to protect current representation from external input.^4^ At the neurobiological level, this dynamic can be understood as a competition between 2 neurotransmitter systems: the norepinephrine (NE) system and the gamma-aminobutyric acid (GABAergic) system.^5^^,^^6^ However, how these 2 neurotransmitter systems jointly play a causal role in this dynamic during event segmentation process remains unknown.

The NE system, originating in the locus coeruleus, modulates neural gain control—the relationship between (sensory) input and output.^7^^,^^8^ An increase in NE levels sharpens gain and strengthens prefrontal working memory circuitry, thereby tightening input–output coupling and facilitating updating of working memory.^9-11^ This implies that even minor changes in the environment could trigger boundary detection and prompt an update of the working event model. The relevance of gain control to event segmentation has already been demonstrated in a previous study; however, that study modulated the entire catecholaminergic system.^12^ In contrast, the GABAergic system exerts inhibitory control that functions as a sensory filter, reducing noise and thereby stabilizing active representations.^13-15^ Moreover, GABA has been linked to working memory capacity, with lower GABA levels associated with reduced capacity.^16^^,^^17^ From the perspective of working event models, increasing GABA levels, and thus working memory capacity, should enable the maintenance of more complex models in working memory. Accordingly, increased GABAergic activity would be expected to reduce the need for frequent updating of the working event model and, in other words, enhance its maintenance.

Simultaneous stimulation of both NE and GABA neurotransmitter systems can be achieved through the application of atVNS.^6^^,^^18^ Applying atVNS to the cymba conchae activates afferent fibers of the auricular branch of the vagus nerve—which innervates this region of the ear and projects centrally to the medullary nucleus of the solitary tract (NTS), thereby increasing input to the NTS.^19^^,^^20^ The NTS serves as a critical relay hub connecting 2 key neuromodulatory pathways. On the one hand, it projects directly to the locus coeruleus—a key brain region for NE release.^19^^,^^21^ An increase in NE release increases gain control and enhances the sensitivity of neurons to (sensory) input.^7^^,^^8^ On the other hand, the NTS also activates additional brainstem nuclei (eg, the parabrachial nucleus), which project to both cortical and subcortical regions, recruiting GABAergic interneurons in the process.^22^^,^^23^ These interneurons subsequently release greater amounts of GABA, leading to a general inhibition of neural signaling and a reduction in neuronal noise.^24^^,^^25^ Therefore, atVNS can simultaneously promote both increased updating and enhanced maintenance of working event models. This dual stimulation of neurotransmitter systems via atVNS provides a unique opportunity to investigate their relative importance in event segmentation. If NE plays a more prominent role in this process, atVNS should enhance the dependency of segmentation behavior on environmental input. Conversely, if GABA is more relevant, atVNS should lead to a stabilization of working event models. Interestingly, previous research on the effects of atVNS on working memory processes suggested that the stimulation tends to enhance the stability of working memory representations, implying a more prominent role of the GABAergic system than the NE system.^18^ Accordingly, the present study utilized atVNS as an experimental manipulation in a single-blind, counter-balanced study to investigate the role of NE- and GABA-mediated mechanisms in event segmentation process.

In order to gain further insights into the stability of the working event model representations, the underlying neurophysiological mechanisms, measured by means of electroencephalogram (EEG), was assessed by employing multivariate pattern analysis (MVPA).^26-28^ This method is well-suited to detect condition-specific representational patterns and to measure how reliably they persist across time. Of particular interest was the change in representational patterns under the influence of atVNS compared to a control condition without active stimulation, as this could provide further insights into whether the stability of the working event model increases or decreases under atVNS. We predicted that, if atVNS increased the stability of the working event model, classification accuracy would decrease relative to the control condition; conversely, if stability was reduced, classification accuracy would increase. Furthermore, source reconstruction was used to capture brain regions contributed to the significant pattern differences detected by MVPA between active atVNS and control conditions. Changes in activity would be expected in regions associated with heightened noradrenergic and GABAergic activity, such as the prefrontal or insular cortices.^29-31^

## MATERIALS AND METHODS

### Participants

A total of 44 healthy participants were initially recruited for the study. Three participants were excluded due to an insufficient number of trials for EEG analysis, and one was excluded because of data recording issues, resulting in a final sample of 40 participants. Their ages ranged from 19 to 35 years (mean age 25.85 ± 4.26 years), with 20 males included. All participants had normal or corrected-to-normal vision. None of the participants reported a history of neurological or psychiatric disorders including substance abuse or dependence, a history of brain surgery, tumors, or intracranial metal implants, or the usage of psychoactive medications. Moreover, due to additional inclusion requirements for the atVNS, none of the participants reported pregnancy, susceptibility to seizures or migraines, or having a pacemaker or other implanted devices. Informed consent was obtained after participants were fully briefed on the study procedures. Upon completion of the experiment, each participant received a financial reimbursement of 35 EUR. The study was approved by the Ethics Committee of the Medical Faculty of TU Dresden and adhered to the Declaration of Helsinki.

### atVNS Stimulation

This study adopted a sham-controlled crossover design, with participants remaining blind to the type of stimulation they received. Each participant completed 2 sessions scheduled at least 1 week apart to minimize carryover effects, with most completing both within 1 month. In the first session, half of the participants received active atVNS stimulation, while the other half received sham stimulation; the order (active-sham vs sham-active) was counterbalanced across participants. Following protocols of previous studies,^32^^,^^33^ stimulation was initiated approximately 20 minutes before the experiment began and continued throughout the completion of the task.

The auricular branch of the vagus nerve was stimulated using the atVNS nextGen research device (tVNS Technologies). Stimulation intensity was individually adjusted between the detection threshold and the pain threshold using a series of 10-second trials. Participants rated their sensation on a 10-point Likert scale (0 = no sensation, 3 = light tingling, 6 = strong tingling, 10 = unpleasantly painful). An increasing series of trials began at 0.1 mA, incrementing by 0.2 mA until a sensation rating of 8 was reached; a subsequent decreasing series repeated the process until the rating fell to 6 or below. This procedure was repeated twice, and the final stimulation intensity was determined by averaging the 4 selected values (2 from each series). The difference between the active atVNS and sham conditions was electrode placement: for active stimulation, the electrode was placed on the cymba conchae (an area rich in vagal afferents), whereas for sham stimulation, it was placed on the earlobe, which lacks vagal innervation^6^^,^^34^^,^^35^ and the stimulation of which thus does not produce cortical or brainstem activation.^36^ Both sessions were administered with the stimulator activated, making them indistinguishable to participants. Stimulation parameters followed established guidelines,^37^ with continuous delivery at 25 Hz and a pulse width of 200–300 ms. To minimize potential cardiac side effects, stimulation was applied exclusively to the left ear.^38^^,^^39^ After each session, participants completed an atVNS aversive effects questionnaire using a 5-point Likert scale to rate potential side effects (eg, headache, neck pain, nausea, facial or neck muscle contraction, stinging under the electrodes, discomfort, and other adverse sensations). Once both sessions were finished, each participant was also asked which session they believed was active session.

### Task

Participants completed an event segmentation task in which they watched a movie and pressed the space key whenever they perceived one event ending and a new one beginning. They were informed that there were no right or wrong responses, as the study relied on their subjective assessments. To help participants familiarize themselves with the task and address any questions, a practice video clip featuring a man preparing a party was presented.^40^^,^^41^ After this practice phase, participants watched the movie *The Red Balloon*^42^ with the instruction of the event segmentation paradigm. This film was selected because of its minimal dialog, frequent situational changes, and clear chronological narrative—features that make it suitable for studying event segmentation.^43-45^ The movie was divided into 4 clips with durations of 463.3, 468.4, 446.2, and 600.6 seconds^45^ with brief breaks of self-chosen duration in between, and the participants could resume the task by tapping the space key. Stimuli were presented and responses were recorded using the “Presentation” software (Neurobehavioral Systems Inc.), and each participant watched the movie once per session. Notably, *The Red Balloon* has been previously scored frame by frame for situational changes (eg, shifts in the location of agents or character interactions) by Zacks et al.^45^ This rating scheme has been applied in several studies^12^^,^^28^^,^^45-51^ and was used in the present study as well.

### E‌EG Recording and Preprocessing

During the event segmentation task, EEG data were collected using elastic EEG caps (EasyCap Inc.) with 60 Ag/AgCl electrodes. The electrodes were secured using the elastic EasyCap system, and conductive gel was applied to each electrode to ensure stable skin contact and maintain low impedance. The reference electrode was placed at Fpz, and the ground electrode was positioned at θ = 58, φ = 78. Signals were amplified using BrainAmp amplifiers (Brain Products Inc.), while keeping electrode impedances below 5 kΩ. EEG data were sampled at 500 Hz and later down-sampled to 300 Hz for offline processing. Preprocessing was carried out using the Automagic pipeline^52^ and EEGLAB.^53^ Flat channels were removed first, and the data were re-referenced to an average reference. Next, the PREP^54^ and EEGLAB “clean_rawdata()” pipelines were applied. Line noise at 50 Hz was filtered out by PREP using a multitaper algorithm, and a robust average reference was calculated after excluding bad channels. Within the “clean_rawdata()” pipeline, a high-pass finite-impusle response (FIR) filter of 0.5 Hz (order 1286, stop-band attenuation −80 dB, transition band 0.25–0.75 Hz) is applied to detrend the data. Noisy, outlier, and inactive channels were detected and discarded. Subsequently, artifact subspace reconstruction (burst criterion = 15^55^) was applied to reconstruct segments with unusually high power; segments that could not be reconstructed were discarded. Moreover, a low-pass filter (40 Hz, sinc FIR filter, order: 86^56^) was applied. Electrooculography artifacts were removed using a subtraction approach.^57^ Independent component analysis, using the “MARA” algorithm,^58^^,^^59^ addressed remaining muscle, heart, and eye movement artifacts by automatic classification and removal. In addition, components with cardiac artifacts were determined and removed using ICLabel.^60^ Finally, missing channels were interpolated using a spherical method.

**Figure 1:**
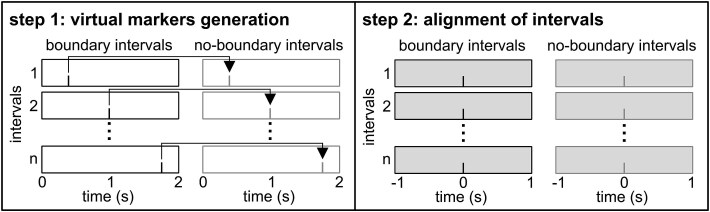
Schematic overview of the creation of no-boundary intervals. Boundary intervals are depicted in black, and no-boundary intervals in gray. In step 1, the data were segmented into 2-second intervals. Each interval containing a boundary (key press) was selected and randomly paired with an interval that did not include a boundary. A virtual marker was then placed within the no-boundary interval at the same relative time point as the key press in its paired boundary interval (left panel). In step 2, data were re-segmented around these virtual markers, allowing for time-locked analysis from −1 to +1 second relative to each marker’s position (right panel).

Further analyses were conducted in FieldTrip.^61^ In addition to exploring the effects of atVNS on the event segmentation processes, particularly around event boundaries, we also aimed at determining whether its influence is task-specific rather than reflecting a general effect of stimulation. To this end, we defined both boundary intervals (BIs), marked by key presses indicating perceived event boundaries, and no-boundary intervals (NBIs), which lacked such key presses. Since NBI lacked real markers, we created virtual markers as shown in Figure 1 by: (1) segmenting continuous data into 2-second epochs, (2) randomly matching NBIs to BIs, (3) projecting the timing of BI key presses onto the matched NBIs, and (4) re-segmenting BI and NBI using the response or the virtual marker, respectively, as central time point zero of the segment. Subsequently, there was an equal number of BI and NBI within participants. EEG data from −1 to +1 second around both real and virtual markers were used for further analysis.^49^

### Multivariate Pattern Analysis

To quantify how neural activity patterns evolved over time across stimulation conditions (active vs sham) and intervals (BI vs NBI), we applied time-resolved MVPA to EEG signals with MVPA-Light toolbox,^27^ following previous studies on event segmentation.^28^^,^^51^ For each trial, EEG data within a time window from −1 to +1 second around key presses were included in the MVPA. For each participant, we applied 2 types of MVPA analyses to both contrasts—stimulation session (active vs sham) and interval type (BI vs NBI). First, a millisecond-resolved binary classifier identified the exact latencies at which scalp topographies reliably differentiated the simulation sessions and interval types, respectively. Second, a temporal-generalization analysis trained the classifier at one time point and tested it across all others, revealing how those condition or interval-specific neural representations emerge, persist, or reactivate over time. We used linear discriminant analysis as the classifier, with performance evaluated using a 10-fold cross-validation repeated 10 times. Unless otherwise specified, default settings from the MVPA-Light toolbox were applied. Classification performance was assessed using both accuracy and the area under the curve (AUC), a non-parametric measure based on signal detection theory. Significant time points with above-chance classification (AUC > 0.5) were identified using cluster-based permutation tests. These tests involved 1000 random permutations and Wilcoxon signed-rank tests at a threshold of *P* = .05. The test statistic for each cluster was the sum of the Wilcoxon values across time points.

### Source Localization (sLORETA)

Standardized low-resolution brain electromagnetic tomography (sLORETA^62^) was applied to the EEG data to localize the brain regions contributing to the distinct neural representation patterns identified across different sessions in the MVPA. We focused on the significant time windows revealed by MVPA to examine which brain areas were involved in the observed differences between active and sham sessions. sLORETA is based on a realistic MNI152 head model and partitions the brain volume into 6239 voxels at a 5-mm resolution, calculating standardized current density at each voxel.^62^ This approach provides a linear, unbiased solution to the EEG inverse problem^63^ and has been validated by studies involving brain stimulation and MRI.^64^^,^^65^ To determine statistical significance, we conducted voxel-wise non-parametric permutation tests (SnPM) with 2000 randomizations. The results section reports significant voxels (*P* < .05) in the Montreal Neurological Institute (MNI) template provided by the sLORETA software that showed differential modulation between sessions.

### Statistics

To examine behavioral effects, we applied a mixed-effects logistic regression model in R (RStudio 2022). To assess whether the likelihood of segmentation rises with the density of situational changes in a movie, the continuous movie was divided into 2-second time bins, and the number of situational changes per bin (0 to 5) was calculated. In the model, fixed effects included the number of situational changes (0 to 5), session (0 = sham, 1 = active), as well as their interaction, with participant included as a random intercept. The binary outcome variable indicated whether a segmentation occurred within each bin (1 = segmentation, 0 = no segmentation), and the model was specified as Segmentation ~ NumChange × Session + (1 | Subs). Linearity of the effect of the number of situational changes on segmentation probability was assessed by comparing the linear specification with a model including a quadratic term. Odds ratios (ORs) were derived from the fixed-effect coefficients. To assess how much the session effect was consistent across participants, we computed the response probability of each subject for each session across all numbers of changes and then calculated their difference with a paired *t*-test. To contextualize nonsignificant findings, we conducted a sensitivity analysis for the within-subject comparison, which indicated that with *N* = 40, the study had 80% power (α = 0.05, 2-sided) to detect a minimum paired effect size of Cohen’s *d* = 0.454 (R package “pwr”).

Stimulation intensity was contrasted between active and sham sessions with a paired *t*-test. Session-related differences in self-reported side-effects were examined first with a repeated-measures analysis of variance (ANOVA) to test for an overall effect. Because preliminary analyses using the mixed-effects logistic regression model (our primary analysis) indicated no main effect of order, this factor was not included in the ANOVA to maintain consistency across analyses. For individual symptom, post hoc comparisons were performed with false-discovery rate (FDR)-corrected paired *t*-tests. Lastly, participants’ ability to identify the active session was evaluated with a one-sample *t*-test on their session guesses.

Statistical significance of MVPA decoding accuracy was assessed using cluster-based permutation testing implemented in the MVPA-light toolbox (MATLAB R2023a). At the group level, a Wilcoxon signed-rank test was used. Clusters were formed by thresholding the *z*-statistic at a cluster-forming threshold corresponding to *P* < .01. Cluster significance was evaluated using 1000 permutations, and clusters were considered significant at a cluster-level threshold of *P* < .05. This procedure controls the familywise error rate across all time points in the time-resolved analyses and across all train–test time pairs in the temporal generalization matrices.

## RESULTS

### The Effects of atVNS Stimulation

The mean stimulation intensity was 2.03 ± 1.15 mA for the active session and 2.24 ± 1.21 mA for the sham session, with no significant difference between them (*t*(39) = 1.03, *P* = .311, Cohen’s *d* = 0.16, 95% CI, −0.62 to 0.20). The repeated-measures ANOVA of self-reported side effects revealed neither a main effect of session (*F*(1,32) = 1.21, *P* = .280), nor an interaction between session and side effect (*F*(7,224) = 1.40, *P* = .208), indicating that stimulation side effects were comparable between active and sham sessions. Table S1 lists the mean ratings for each of the 8 symptoms together with FDR-corrected paired *t*-tests. None of the individual comparisons reached significance. Besides, participants could not reliably identify the active session, as their guess did not differ from chance (*t*(37) = 1.66, *P* = .106, Cohen’s *d* = 0.27, 95% CI, −0.03 to 0.29), confirming that blinding was effective.

### Behavioral Results

**Figure 2:**
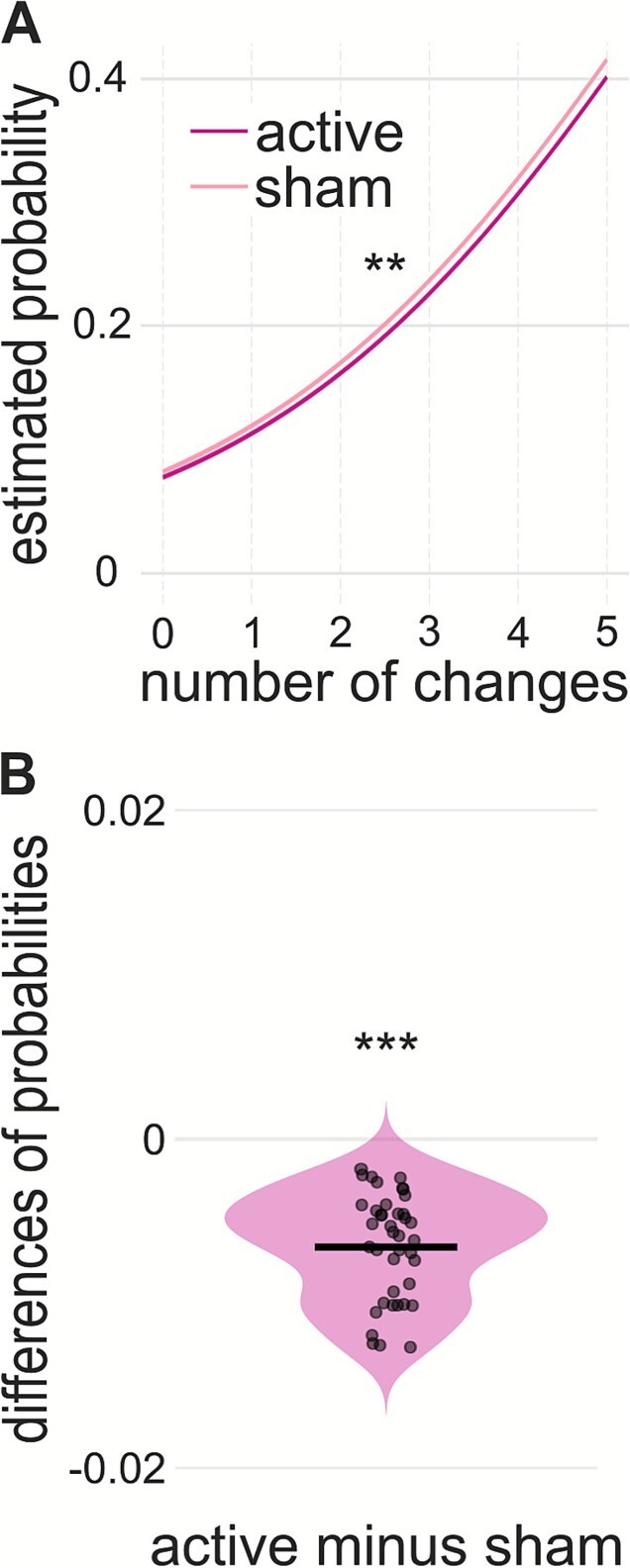
Behavioral results of mixed-effects logistic regression. (A) Model predicted probability of segmentation (y-axis) with 2 predictors-situational change counts per 2 seconds and session-with separate curves for active atVNS (magenta) and sham (pink). (B) Violin plots display the distribution of within-subject differences in predicted segmentation probabilities (active minus sham). Each dot represents one subject, and the horizontal black bar marks the mean difference. Overall, active atVNS reduced the likelihood of segmentation across levels of situational change, consistent with increased stability of the working event model. atVNS, auricular transcutaneous vagus nerve stimulation; VNS, vagus nerve stimulation.

The mixed-effects logistic regression with 2 predictors *number of changes* and *session* revealed a significant intercept (*z* = −17.82, *P* < .001, odds-ratio (OR) = 0.12, 95% CI, −2.31 to −1.86), significant coefficients for both the number of changes (*z* = 33.18, *P* < .001, OR = 1.51, 95% CI, 0.39-0.44) and session (*z* = −2.74, *P* < .01, OR = 0.94, 95% CI, −0.11 to −0.02), while the interaction between these 2 factors was not significant (*z* = 0.05, *P* = .96, OR = 1.00, 95% CI, −0.03 to 0.04). The variance of the subject level random intercept was 0.536, indicating moderate between-subject variability in segmentation probability. As shown in Figure 2A, segmentation probability increased with the number of situational changes across both sessions, with an overall difference in segmentation likelihood between sessions. Including a quadratic term for the number of situational changes significantly improved model fit (χ^2^ = 266.35, *P* < .001), indicating a nonlinear relationship on the logit scale; however, the direction of the effect, the session effect, and the absence of interactions were unchanged. Figure 2B displayed the differences of each participant for the estimated response probability with active minus sham. These values were consistently lower in active sessions, and the paired *t*-test indicated a significant difference (*t*(39) = 12.896, *P* < .001) between the 2 sessions.

### Neurophysiological Results

**Figure 3:**
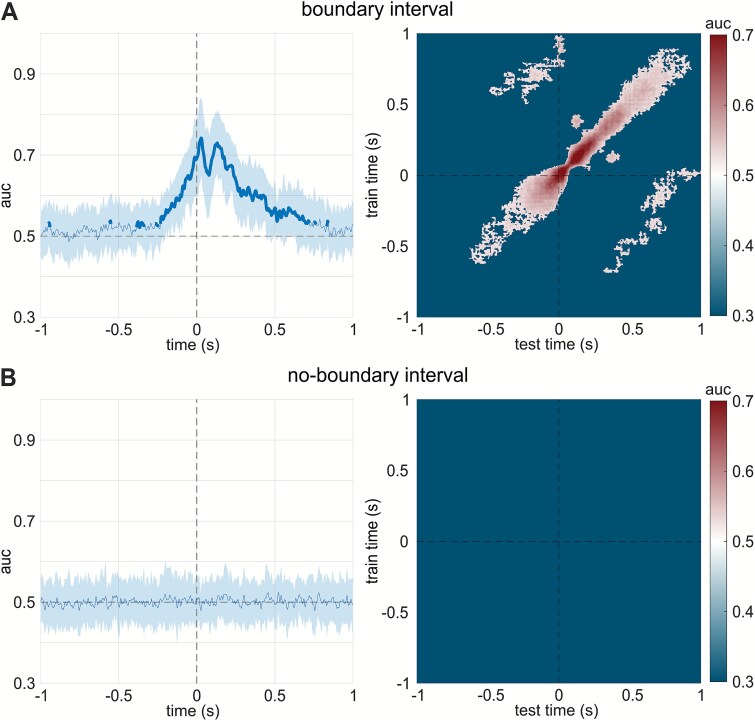
MVPA results comparing active and sham sessions for both boundary and no-boundary intervals. (A) Boundary interval. Left: time-resolved classification performance expressed as area under the curve (AUC); bold lines mark above change periods, with shading area reflecting the standard deviation across participants. Right: temporal generalization matrix; color encodes AUC, with brighter regions indicating greater between-session discriminability across train-test times. (B) No-boundary interval. Same layout; neither the time-resolved nor the temporal generalization analysis shows significant classification. These results indicate that atVNS selectively modulated neural representations during event boundaries, rather than producing a global session difference. atVNS, auricular transcutaneous vagus nerve stimulation; MVPA, multivariate pattern analysis.

Figure 3A shows the across-time binary classification performance for BI, comparing sham and active sessions. MVPA identified a significant discrimination window from approximately 230 ms before to 723 ms after the event boundary (mean AUC = 0.612; range 0.523–0.768), forming a significant cluster (cluster mass = 1410.372, cluster size = 287, cluster-level *P* < .001). Furthermore, the temporal generalization MVPA for BI established that roughly 11.37% of classification pairs yielded significant AUC values (mean AUC = 0.555; range 0.50–0.768), forming a significant diagonal cluster from approximately 263 ms before to 727 ms after the event boundary (cluster mass = 120421.493, cluster size = 28 781, cluster-level *P* < .001). Moreover, off-diagonal generalization emerged around 500 ms before the boundary and gradually tapered off by 50 ms after the boundary, indicating stable cross-temporal neural pattern generalization. Off-diagonal clusters were defined as contiguous regions of significant decoding values located outside the main diagonal of the temporal generalization matrix. These clusters were evaluated using the same cluster-based permutation test across the entire matrix. Two significant off-diagonal clusters were identified: one in the upper-left quadrant (cluster mass = 2278, cluster size = 7240.283, cluster-level *P* < .001) and one in the lower-right quadrant (cluster mass = 2282, cluster size = 7278.479, cluster-level *P* < .001). In contrast, classification performance across time for the NBIs (Figure 3B) did not differ significantly from chance level. Similarly, the temporal generalization MVPA for NBI showed no classification performance above chance.

**Figure 4:**
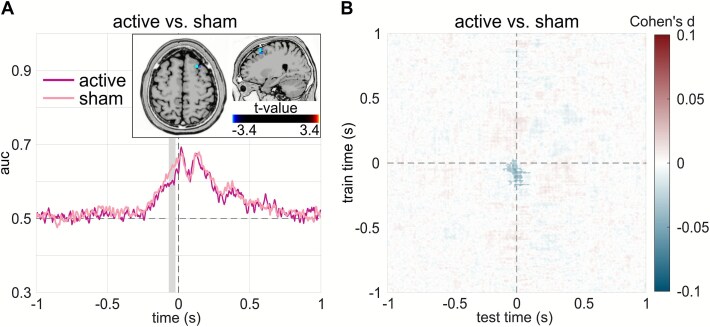
Session-wise comparison of MVPA decoding accuracy with the corresponding sLORETA result. (A) Time-resolved BI–NBI decoding (AUC): magenta curve for active session and pink curve for sham session; gray shading highlights time periods with a significant AUC difference between sessions; sLORETA maps show brain regions exhibiting significant active-sham contrasts within the gray-marked window. (B) Temporal generalization BI–NBI decoding (AUC): Cohen’s *d* effect size map for active vs sham across train-test matrix; the color scale indicates effect magnitude within cluster-corrected significant regions. Active atVNS was associated with reduced BI–NBI decodability, consistent with increased stabilization of event representations prior to segmentation. atVNS, auricular transcutaneous vagus nerve stimulation; BI, boundary interval ; NBI, no-boundary interval; MVPA, multivariate pattern analysis; sLORETA, standardized low-resolution brain electromagnetic tomography.

Additionally, MVPA was also calculated between BI and NBI separately for active and sham sessions. Importantly, the subsequent comparison revealed that active session had a significant lower AUC compared with sham session within a time window before segmentation (from −67 to −23 ms, cluster mass Tsum = −35.62, cluster-level *P* = .04), as showed in Figure 4A. In the temporal generalization matrix (Figure 4B), decoding differences between active and sham sessions were also observed. A significant diagonal cluster indicated reduced AUC in the active session relative to sham, spanning approximately −200 to 0 ms for the train time and − 100 to 100 ms for the test time (cluster mass Tsum = −3378.33, cluster-level *P* = .04). Source localization was performed on the time windows in which BI vs NBI decoding accuracy (AUC) differed significantly between sham and active sessions. As shown in Figure 4A, during the significant time window before segmentation, activation in the right middle/superior frontal gyri (BA6/BA8) was significantly lower in the active session than in the sham session (see supplementary Table S2 for details on the coordinates). Further, we also compared the AUC values of the temporal generalization MVPA between sessions. These analyses revealed significant differences (Figure 4B) between active and sham sessions (active < sham) along the diagonal, spanning approximately from −200 to 0 ms for train time and −100 to 100 ms for test time.

## DISCUSSION

The current study aimed to investigate the relevance of the NE and GABAergic systems in event segmentation by using atVNS, drawing on the EST.^1^^,^^2^ According to EST, the *working event model*—a representation of the current situation—is the central element in event segmentation. Working event model representations are updated when predictions are violated by ongoing input and maintained when they are matched. This update-maintenance dynamic is differentially modulated by NE and GABAergic systems.^5^^,^^6^ We examined these effects at both the behavioral level and neurophysiological level using EEG-based MVPA and source localization.

The behavioral results indicated an overall reduced likelihood of event segmentation behavior under the influence of active atVNS compared to sham stimulation (Figure 2A). This reduction was uniform across levels of environmental change, as reflected by the absence of an interaction between stimulation mode and the number of environmental changes. This suggests that the working event model exhibits greater stability under atVNS, and event boundaries are therefore less likely to be set. Interestingly, this result aligns with a previous study examining the stability of working memory representations under atVNS.^18^ That study demonstrated that atVNS was associated with a tightening of the working memory gate via GABAergic modulation—rather than NE system —thereby restricting the entry of new information and making updating processes less likely and harder to initiate.^18^ Consistent with this working memory gating account, the present findings further indicate increased stability of the working event model in working memory under atVNS relative to sham, reflecting a bias toward model maintenance rather than updating. Importantly, because neuromodulatory state was manipulated experimentally, the present findings extend earlier observational accounts by demonstrating that changes in neuromodulatory activity can directly influence event segmentation.

Changes in the representations of the working event models were also reflected in the neurophysiological findings of the MVPA. Specifically, around indicated event boundaries, classifiers reliably distinguished active atVNS from sham stimulation (Figure 3A), indicating that atVNS selectively altered segmentation-related processing. Similarly, in the temporal generalization MVPA, decodability was primarily observed along the diagonal, with additional significant clusters off the diagonal linking time periods before the event boundary to those after it. Notably, the presence of off-diagonal decodability also indicated that the influence of prior event segments on the current one is modulated by atVNS.^66^ Intriguingly, in the no-boundary interval, no distinguishable differences in mental representations between active atVNS and sham conditions were observed (Figure 3B), suggesting that the effects of atVNS modulation were specific to event segmentation rather than reflecting general differences between sessions. Crucially, before the end of events, in the active session, there was reduced decodability between boundary and no-boundary intervals relative to sham (Figure 4A). This reduction parallels the behavioral decrease of segmentation likelihood and is consistent with a GABAergic stabilization of working event models.

Of note, atVNS also engages the NE system: continuous atVNS stimulation is thought to bias the LC toward a higher tonic mode, dampening phasic, boundary-linked responses, and reducing sensitivity to small prediction error-effects that would likewise reduce segmentation.^6^^,^^10^^,^^67^ In this way, also a tonic NE mode could contribute to more stable working memory representations.^11^ Nevertheless, several observations are more consistent with a predominantly GABAergic account. First, prior work showed that atVNS closed the working memory gate by GABAergic modulation rather than NE mechanisms.^18^

Given that a working event model—the key element of the event segmentation process—is itself a working memory representation, it’s likely modulated in the same way. Second, our earlier study showed that raised NE boosted the gain control, which made people more likely to mark event boundaries—the opposite of what we see here.^68^ Third, if atVNS simply elevated tonic NE arousal, baseline enhancement effects would be expected throughout the task, both at boundary and no-boundary intervals. The absence of effects during no-boundary intervals was inconsistent with a global NE-mediated arousal shift and instead pointed to task-specific inhibitory gating. Taken together, the data are more consistent with a GABAergic modulation account.

The assumption that working memory processes were particularly affected was also reflected in the results of the source localization analysis. Source estimation revealed decreased activity in regions encompassing the right superior and middle frontal gyri (Figure 4A) under atVNS compared to sham stimulation, prior to the setting of an event boundary. These 2 brain regions both belong to the dorsal lateral prefrontal cortex, which is suggested to play a key role in executive control and working memory.^69-71^ This decreased source estimated activity accords with the interpretation of stabilized mental representations, as this region has been implicated in the active maintenance and top-down modulation of working memory contents. Moreover, GABAergic modulations in prefrontal regions have been shown to be related to working memory processes.^16^^,^^17^

It is important to note that the present study did not directly measure GABAergic or noradrenergic activity. Moreover, although EEG provides high temporal resolution, its limited sensitivity to subcortical structures constrains direct inferences about neurotransmitter dynamics. Accordingly, while the observed behavioral and neurophysiological findings are consistent with a GABAergic modulation account, definitive causal conclusions cannot be drawn. In addition, the present findings were obtained using a relatively constrained stimulus (a single narrative film), and their generalizability to more complex events remains to be established. Future studies incorporating direct neurochemical measurements (eg, magnetic resonance spectroscopy)^72^^,^^73^ or pharmacological interventions with more diverse stimulus contexts will be essential to disentangle the respective contributions of GABA and NE to event segmentation.

In summary, the present study provides converging behavioral and neurophysiological evidence that atVNS modulates event segmentation by stabilizing mental representations in working memory. The reduced probability of event boundary setting under atVNS, along with the observed decrease in decoding accuracy and reduced activation in working memory-related cortical regions, supports the hypothesis that GABAergic modulation plays a key role in maintaining the working event model. These findings extend previous work on atVNS and working memory by demonstrating its influence on dynamic, ecologically relevant cognitive processes such as event segmentation.

## Supplementary Material

supplemental_material_R1_pyag002

## Data Availability

The data underlying this article will be shared on reasonable request to the corresponding author.
