# Supplementary material for: Auricular transcutaneous vagus nerve stimulation stabilizes event segmentation through modulation of working memory representations
Source: Int J Neuropsychopharmacol. 2026 Jan 23;29(2):pyag002. doi: 10.1093/ijnp/pyag002 (PMC12911928; doi:10.1093/ijnp/pyag002)
Supplement: supplemental_material_R1_pyag002 [file supplemental_material_r1_pyag002.docx]

**Supplemental Material**

**Auricular Transcutaneous Vagus Nerve Stimulation Stabilizes Event Segmentation Through Modulation of Working Memory Representations**

Xianzhen Zhou, Foroogh Ghorbani, Veit Roessner, Bernhard Hommel, Astrid Prochnow, Christian Beste

Supplementary Table 1. Subjective side effect ratings for active atVNS and sham stimulation sessions

|  | Active | Sham | T value | P value |
| --- | --- | --- | --- | --- |
| Headache | 1.15 (0.36) | 1.21 (0.42) | -0.63 | 0.72 |
| Neck pain | 1.27 (0.45) | 1.36 (0.65) | -0.90 | 0.59 |
| Nausea | 1.03 (0.17) | 1.03 (0.17) | 0 | 1.00 |
| Muscle contraction in face or neck | 1.33 (0.54) | 1.39 (0.75) | -0.47 | 0.73 |
| Stinging sensation | 1.70 (0.81) | 1.61 (0.75) | 0.90 | 0.59 |
| Burning sensation | 1.36 (0.60) | 1.15 (0.36) | 2.03 | 0.24 |
| General discomfort | 1.48 (0.67) | 1.33 (0.54) | 1.97 | 0.24 |
| Other sensations and/or aversive effects | 1.24 (0.50) | 1.12 (0.42) | 1.07 | 0.59 |

*P value after correction with FDR

Supplementary Table 2. Voxel-wise summary of sLORETA results for the active vs. sham contrast during the pre-boundary time window from -67ms to -23ms

| Contrast | X (MNI) | Y (MNI) | Z (MNI) | Test statistic | Brodmann area | Structure |
| --- | --- | --- | --- | --- | --- | --- |
| Active < Sham | 25 | 20 | 55 | -3.56 | 8 | Superior Frontal Gyrus |
|  | 25 | 20 | 60 | -3.44 | 6 | Middle Frontal Gyrus |

Note: voxel-wise sLORETA statistics were thresholded at p < 0.05 (one-tailed, t < -3.38), reflecting the a priori directional hypothesis given the active session showed reduced decoding related activity compared with the sham session.
